# Bridging Cultures in Rural Health: A Review of Long Road from Quito and Interview with Author Tony Hiss

**DOI:** 10.4269/ajtmh.19-0294

**Published:** 2019-07

**Authors:** Barnett L. Cline

**Affiliations:** Professor Emeritus; Tulane Universityy New Orleans, Louisiana; E-mail: barnett.cline@gmail.com

“I’m going to die,” Alfredo told Dr. David Gaus after the seven-year-old was bitten by a pit viper. “You will be fine,” the boy was reassured. But during the five-hour drive to Quito, Alfredo perished. David Gaus had to tell Alfredo’s mom.

Twenty-eight-year-old Vicente died in the waiting room of a hospital in Quito because the cause of his respiratory failure—acute agricultural exposure to organophosphates—was unfamiliar to urban physicians. And 43-year-old Gustavo, his femur fractured in a road accident, bled to death before reaching Quito.

In the late 1990s, these and other tragedies forced David Gaus to rethink his dream of building a primary care clinic in rural Ecuador and to envision something bigger. But now let us step back. How, 15 years earlier, the Notre Dame accounting major landed in Ecuador in the first place is a story in which Father Theodore “Ted” Hesburgh, Notre Dame’s president, played a central role. When Gaus confessed ambivalence over a future spent poring over numbers in his home state of Wisconsin, Hesburgh’s recommendation that David work with street kids in Quito fueled a fierce determination to return to Ecuador as a physician. How David accomplished this is a story as improbable as it is intriguing. As are specific events in Gaus’s life: meeting his Ecuadorian wife Elizabeth; bringing her to Wisconsin; having children; returning to Ecuador as a family after David completed medical school and tropical medicine training, and a family medicine residency; and, finally, in 1998, creating the nonprofit Andean Health and Development (AHD) with Hesburgh as its founding chair.

It was at this point I joined AHD’s advisory board and happily accepted an invitation to attend its inaugural board meeting in Quito. Thirty-six hours after my arrival, Father Ted’s plane would also touch down. I spent the night at the Gaus’s home overlooking sprawling Quito, and early the next morning accompanied David to his storefront clinic in Pedro Vicente Maldonado (PVM), a rural community named after an 18th-century Ecuadorian cartographer who designed an overland route from Quito to the Pacific coast. Descending through dramatic mountain vistas while traveling in a westerly direction, we were nearing the town of San Antonio when David stopped at a monument marking where the road crossed the equator. As I realized I was standing on this real but imaginary 25,000-mile line, I experienced a visceral jolt. On the other hand, what lay beyond Ecuador’s equator is what captured my interest for decades to follow.

Before we returned to Quito’s airport to greet “Father Ted,” bilingual, bicultural David walked me through the streets of PVM as he explained why this community, which had virtually no formal medical care, was an ideal locale in which to build a primary care clinic. The same idea had long incubated in the minds of AHD cofounders Tom Chiller and Erik Janowski, two of David’s closest friends from Notre Dame and Tulane Medical School. David then shared his ultimate vision to provide not just a clinic but a hospital. How could medical caregivers truly gain the respect and trust of a community if they could not meaningfully respond to urgent medical crises such as those afflicting Alfredo, Vicente, and Gustavo? High-quality rural hospitals, he argued, must become an essential part of the World Health Organization initiative “Health for All by the Year 2000”. As we continued to walk the dusty unpaved streets of PVM, David described recent discussions with the mayor, farming cooperatives, and other entities that might join in creating a small, self-sustaining hospital to serve roughly 50,000 largely impoverished residents of the region.

Now, 21 years later, comes *Long Road from Quito: Transforming Health Care in Rural Latin America*, a book that captures what David Gaus and AHD have accomplished. Tony Hiss, who has previously authored 13 other books, was formerly a long-time staff writer at the *New Yorker* and is presently based at the New York University. Far more than an inspirational portrait of David Gaus, Hiss’s work skillfully interweaves storytelling and content to illustrate fundamental concepts of global public health and its ongoing evolution.

Readers of *Long Road from Quito* will discover how AHD opened Hospital PVM in 2000, and later created the larger Hospital Hesburgh in heavily populated Santo Domingo, in both cases achieving financial sustainability through innovative local partnerships and 21st-century management practices. In addition, both hospitals and their outpatient clinics are entirely staffed by Ecuadorians. Having already trained 76 residents, the two hospitals also embody two further pillars of AHD’s mission: training Ecuador’s rural health leaders of the future and conducting high-quality, relevant research. Accordingly, the World Health Organization, the Pan American Health Organization, and Ecuador’s Ministry of Health recently recognized AHD for having launched a model program of rural health care.

*Long Road from Quito* also depicts a challenge far greater than geographic isolation—namely, how rural populations differ from urban populations in their perception of health and disease. Such insights are essential not just in Ecuador but in much of the world, including industrialized nations.

Last but not least, Hiss’s book rightfully credits Gaus’s visionary Ecuadorian partner, physician Diego Herrera, a dedicated Ecuadorian staff, and an engaged and passionate advisory board. This is a book with lessons for many: from practitioners in developing countries, to students of public health, global health, and tropical medicine, to specialists in medical anthropology and modern social justice, to global philanthropists.

## Five Questions For Tony Hiss

**The diverse subject matter of your previous books made me wonder what motivated you to take on this project. Was it partly to educate yourself about global health?**

When I first heard about David Gaus from friends, I got intrigued by this inventive, innovative person. After meeting and traveling with Gaus in Ecuador, I became deeply fascinated by a concept I was newly exposed to: the idea of health as something more than simply the absence of disease, much as peace is more than simply the absence of war but the beginning of a civilization. *Long Road from Quito* was a project that took several years and multiple trips to Ecuador. I came to understand health as part of a “landscape of expectations.” Many Americans, unfortunately not all, can look forward to a lifetime of health, something they can rely on and will return to after an illness or operation. But for most Ecuadorians, this is far from the case. I also wanted to learn how Gaus, and the nonprofit AHD, was changing this landscape in rural Ecuador, and perhaps in other parts of Latin America.

**What insights or discoveries about David most stood out for you?**

After spending considerable time with David, a Wisconsin native, I realized he had become trilingual—he could speak English, Spanish, and he had learned to communicate effectively in a rural Ecuadorian society. I also observed, with admiration and affection, that David’s unique combination of passion and compassion permitted him to persist in his efforts, often against long odds that would have defeated many others. It became clear to me that David is a remarkable person who seems remarkably unremarkable; he is open, engaging, modest, fun to be around, and he treats everyone the same, with respect.

**In your book, *In Motion: the Experience of Travel*, you introduce the idea of deep travel. What is deep travel, and did you experience it in Ecuador?**

Deep travel is about opening yourself up to experience everything a place has to offer—all channels are open and operating. Ecuador, with its lush environment, welcoming people, and much poverty, opened up multiple avenues of deep travel for me. Spending countless hours with Gaus permitted me to “travel” into the previously unknown lives of Ecuadorians.

**Can you comment on the dynamic relationship between Diego Herrera and David, and between David and the AHD advisory board?**

Diego, David’s partner, is a brilliant physician who himself straddles two worlds of Ecuador, and I greatly admire his skills. He came from a family of a past president, but lived most of his life in the countryside. He is deeply devoted to patient care, and he and David make a terrific team—his knowledge of Ecuador and its culture, and his powerful administrative skills make him especially effective in dealing with Ecuadorian organizations and in attracting high-potential family medicine residents. As for AHD’s advisory board, I find its members exceedingly close-knit, and their deep affection for David and Diego is clear. They are a smart and diverse group. The board has been effective at pulling and pushing, always helping overcome obstacles and, when necessary, reinventing AHD. The positive role of Laura Dries, AHD’s chief operating officer, is another great plus.

**Happily you were able to visit Father Ted, a man who advised presidents and popes. Did your brief meeting in his 13th floor library office enlarge your appreciation of not only his contribution to AHD but of his legacy?**

Father Ted is the kind of mentor one dreams of finding, one who reaches out to the best in you, and then nudges you to do even better. I learned that Father Ted was deeply committed to AHD, and I believe that this nonprofit, and Hesburgh Hospital in Santo Domingo, Ecuador, will be an essential part of his legacy. Andean Health and Development was, in a real sense, his final project.

